# Privacy preserving protocol for detecting genetic relatives using rare variants

**DOI:** 10.1093/bioinformatics/btu294

**Published:** 2014-06-11

**Authors:** Farhad Hormozdiari, Jong Wha J Joo, Akshay Wadia, Feng Guan, Rafail Ostrosky, Amit Sahai, Eleazar Eskin

**Affiliations:** ^1^Department of Computer Science, ^2^Bioinformatics IDP, ^3^Department of Mathematics and ^4^Department of Human Genetics, University of California, LA 90095, USA

## Abstract

**Motivation:** High-throughput sequencing technologies have impacted many areas of genetic research. One such area is the identification of relatives from genetic data. The standard approach for the identification of genetic relatives collects the genomic data of all individuals and stores it in a database. Then, each pair of individuals is compared to detect the set of genetic relatives, and the matched individuals are informed. The main drawback of this approach is the requirement of sharing your genetic data with a trusted third party to perform the relatedness test.

**Results:** In this work, we propose a secure protocol to detect the genetic relatives from sequencing data while not exposing any information about their genomes. We assume that individuals have access to their genome sequences but do not want to share their genomes with anyone else. Unlike previous approaches, our approach uses both common and rare variants which provide the ability to detect much more distant relationships securely. We use a simulated data generated from the 1000 genomes data and illustrate that we can easily detect up to fifth degree cousins which was not possible using the existing methods. We also show in the 1000 genomes data with cryptic relationships that our method can detect these individuals.

**Availability:** The software is freely available for download at http://genetics.cs.ucla.edu/crypto/.

**Contact:** fhormoz@cs.ucla.edu or eeskin@cs.ucla.edu

**Supplementary information:** Supplementary data are available at *Bioinformatics* online

## 1 INTRODUCTION

Detecting relatives from genetic data is one of the fundamental problems in genetics. As genotype-chip technologies reduce the cost of collecting genetic data for each individual, many personal genomic companies provide various services. One such service is the identification of relatives using genetic data. The underling idea of this service is to collect genotypes of different individuals and to store their data in a database. Then, the genotype for each pair of individuals is compared and any pair of individuals that appear to be genetically related are notified of a match.

Unfortunately, the current version of this service provided by all companies requires individuals to share their genetic data with a trusted company.

Homer *et al.* (2008) already raised many privacy issues by showing that we can detect the existence of an individual in a pool of individuals when the minor allele frequency is available. Thus, the disease status of any individual involved in a GWAS might be exposed to the public. Furthermore, Sankararaman *et al.* (2009) extended the work (Homer *et al.*, 2008) and showed that with access to thousands of variant summary statistics is enough for detecting the existence of an individual in a pool.

Recently, He *et al.* (2013) have proposed a secure method for detecting the genetic relatives using genotype data. This method uses the ‘fuzzy’ encryption (Dodis *et al.*, 2008; Ishai *et al.*, 2011). The ‘fuzzy’ encryption is very similar to the traditional encryption and decryption protocols where each individual has a public key and a private key. Public key for each individual is accessible by all the other individuals and the private key for each individual is hidden from all the other individuals. In the traditional protocol, we use the same private key to decrypt the message that was used to encrypt the message in the first place. However, in the ‘fuzzy’ encryption the two keys should be only close but not necessarily the same. Thus, an individual can detect the genetic relatives by downloading the available public key for all other individuals and compare their public key with his private key. They show if two individuals are genetically related their secure method can detect them while not leaking any information. Moreover, this method is designed such that individuals who are not related to others will not obtain any information. A drawback of this approach is that it can only be applied to common variants.

We propose a novel encoding mechanism that convert each individual’s haplotypes to a set of integer values such that the comparison between two sets approximate the genetic comparison between the two individuals where each individual has access only to its own variants list. The main innovations of our approach compared to He *et al.* (2013) is that we use a novel encoding which allows for us to utilize all variants in an individual’s genome. This is challenging because many of the variants have not yet been discovered. In addition, our cryptographic scheme uses list decoding which has some advantages to other approaches for fuzzy encryption.

We use both simulated and real data to show the utility of our method. We generated series of family relationships using the 1000 genomes data as the founder of the population. Then, we randomly generated offsprings for different generations. With the simulated data, we show that our secure protocol could detect up to fifth degree cousins. However, the previous method (He *et al.*, 2013) can only detect up to third degree cousins. Furthermore, we use Luhya in Webuye (LWK) population from the 1000 genomes data (1000 Genomes Project Consortium, 2010, 2012) that contains cryptic relationships to show that method could detect these cryptic individuals.

## 2 METHODS

### 2.1 Overview

**Fig. 1.**
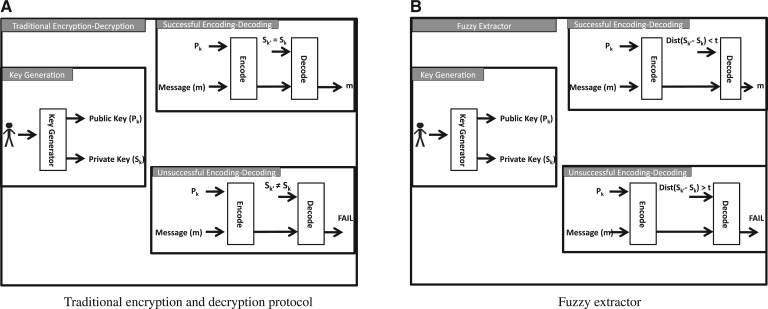
In traditional encryption and decryption protocol, each individual generates two codes using the key generation process. The public key (*P_k_*) is accessible by every one, and the private key (*S_k_*) should be kept secret. In order to send a secure message to a sender we will use the public key available by the sender to encode the message. Then, the receiver will use the secret key (private), which was generated for the sender with the public key in the key generation process, to decrypt the message as shown in panel (**A**). The Fuzzy extractor is similar to traditional encryption and decryption protocol with one major difference, that the private key to decrypt the encrypted message has to be close to the original private key, which was generated in key generation process, and not necessary the same key as shown in panel (**B**)

Our method uses the ‘fuzzy’ encryption, which is a new method in the field of cryptography (Dodis *et al.*, 2008; Ishai *et al.*, 2011). The ‘fuzzy’ encryption is similar to the traditional encryption and decryption protocols where each individual has a public key and a private key. The public key for each individual is accessible by all other individuals and the private key for each individual is hidden from all other individuals. In a traditional protocol to decrypt the message we use the same private key that was used to encrypt the message in the first place as shown in Figure 1A. However, in ‘fuzzy’ encryption, decryption is possible only if the Hamming distance between the two keys is less than a predefined threshold ‘*t*’ as shown in Figure 1B. The ‘fuzzy’ decryption terminates successfully if the Hamming distance between the keys is <‘*t*’ and it fails otherwise. Mostly, the keys used in ‘fuzzy’ encryption are in form of extremely long vectors which are sparse and the sparsity allows us to compute the Hamming distance efficiently using ‘fuzzy’ encryption.

Fuzzy extractors can be used to implement secure comparison of sets of a fixed size (number of elements in a set) which is the basis of our approach to private relative identification. The secure comparison of sets works as follows. Each individual has a set of elements which is private to the individual. Using the cryptographic protocol based on fuzzy extractors, each individual is able to identify which other individuals have a set with at least ‘*t*’ elements in common. The way the protocol works is that each individual releases some public information referred to as a ‘secure sketch’ and then individuals compare their sets against the sketches of others. The individual can recognize if the sets of the two individuals contain at least ‘*t*’ common elements.

The way secure set comparison is implemented using fuzzy extractors is that the private keys that are generated encode the membership of each element of the set. We consider all sets contain *k* elements, each of which is binary vectors of length *m*, then there are a total of 2*^m^* possible elements. The private keys are binary vectors of length 2*^m^* with *k* ‘1’s encoding which element exists in an individual’s set. We use fuzzy extractors to generate public keys for these private keys where the threshold for decryption is 2k−t. Any pair of private keys which have Hamming distance <(2k−t) are correspond to sets that have at least ‘*t*’ elements in common. Any pair of private keys that have Hamming distance of >(2k−t) will have <‘*t*’ elements in common. Each individual can release their public keys and other individuals can detect if their sets have at least ‘*t*’ elements in common by attempting to decrypt the public key using their private key.

In this work similar to previous work (He *et al.*, 2013), we use the fuzzy extractor to compute the symmetric set difference as a black box. Our goal is to encode the two haplotypes (diploid genome) for each individual to a set such that the symmetric set difference between individuals corresponds to the genetic similarity between the two individuals. In the previous method (He *et al.*, 2013), only the common variants are used and assumed the list of variants between all the individuals are the same, as a result we convert the haplotypes to a set by considering non-overlaping segments. Thus, the symmetric set difference between the generated sets can approximate the hamming distance between their haplotypes. However, in our work we want to utilize the rare variants and relax the assumption that all individuals have access to the list of all the variants between all the individuals. In this work each haplotype is compared against the reference genome and the positions where they differ are marked as ‘1’ and the rest are marked as ‘0’. Thus, individuals that are related have more positions in the haplotype marked similarly as compared to the unrelated individuals. Using the encoded genome we generate ‘sketch’ that contains private information and is used as the private key. From the sketch we generate the ‘secure sketch’ and use it as the public key. In order for two individuals ‘A’ and ‘B’ to detect if they are related or not, individual ‘A’ compares its private sketch with the secure sketch obtained from individual ‘B’. If the two individuals are related the ‘fuzzy’ encryption method terminates successfully, if not the program fails.

We need to show our method is secure as each individual release a public key that is generated from each genome that contains private data. We need to show the amount of information obtained from public key is small relative to the total amount of data in each genome. We use entropy to measure the amount of information. Entropy is a known quantity to measure the amount of information in a data and entropy is an additive quantity. Thus, in order to show our method is secure we have to show the entropy in the human genome is much larger than the entropy in the public key (sketch). The entropy in ‘fuzzy’ encryption is bounded by $\frac{t^{2}}{s}$ where ‘*t*’ is the number of elements that are in common between the sets and *s* is the number of elements in each set. Intuitively, this value corresponds to the strength of an encryption. If there are 100 bits of entropy remaining, a brute force approach to identify the set would require the same effort to crack 100-bit encryption. As long as this number is >100 bits, the protocol is relatively secure.

### 2.2 Estimating genetic relatives by comparing sets

There exist a series of methods to detect the relatedness among different individuals and even build the family tree using the Identity by descent (IBD) (Li *et al.*, 2010; Stevens *et al.*, 2011; Wang, 2011). In this section we describe a simple method to approximate the relatedness using the haplotype data which can be used to build a secure protocol.

We assume that we have *N* individuals and we have access to each individual’s variants and the reference genome. In our method we only consider single-base variants which include both common and rare variants. Furthermore, we assume we have access to the phased haplotypes of each individual, in the case we have unphased haplotypes, we can phase them by using the existing methods (Browning and Browning, 2007; Li,Y. *et al.*, 2010; Scheet and Stephens, 2006; Stephens and Scheet, 2010), we phased the individuals using a reference dataset of individuals which did not contain any individuals that are related to the ones we are phasing. We convert the two haplotypes for each individual to a single set such that the set comparison between the two individuals’ haplotypes can estimate the genetic relatedness. In our method, unlike the previous method, the list of all the variants is not the same between all the individuals. Thus, we need to convert each individual’s haplotypes to a binary string such that the hamming distance between the two strings estimates the similarity between the two individuals. Furthermore, the variants that occur in the same positions in the haplotype should be compared against each other. Thus, we use the reference genome to align the variants such the same variants are compared. We convert each individual genome (donor) to binary genome by comparing each donor genome to the reference genome, we convert each position to ‘0’ when there exists no variants between the donor and the reference genome and otherwise ‘1’. We partition each binary genome to non-overlapping segments of 30 000 bp. We generate a set for each individual such that each element of the set contains the segment data (string of length 30 000 which represents the binary genome of that segment) and the segment position. We compute the summation of the binary value of the segment position and the segment data and store the computed value in a set. In order to compute the summation we used the arithmetic addition operation for binary numbers. More formally, let *H_i_* indicates the *i*-th individual binary haplotypes where $H_{i}=\{H_{i}^{1},H_{i}^{2}\}$ such that $H_{i}^{1}$ and $H_{i}^{2}$ represent the first and second haplotypes, respectively, for *i*-th individual. In our model we consider two haplotypes for each individual as we assume we are dealing with diploid genomes (two copies of each chromosome). Moreover, $H_{ij}^{\left\{1,2\right\}}\in {\left\{0,1\right\}}^{30 000}$ represent the *j*-th segment of the *i*-th individual’s binary haplotype. We use *S_i_* to indicate the set for *i*-th individual and *s_ij_* to indicate the *j*-th element of the set *S_i_* representing the *j*-th segment of genome.

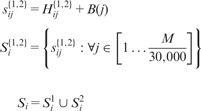

where B(.) denotes the binary representation of an integer number and *M* denotes total number of base pair in each genome, in the case of human genome *M* = 3 billions.

If the distance score between two individuals is <‘*t*’ we consider them as related individuals and if the distance score is >‘*t*’ we consider them as unrelated individuals. We assume the value of ‘*t*’ is computed using a training set where the true relationship between each pair of individuals is known.

In order to compute the number of matched segments between two individuals, we count the number of shared haplotypes for each segment between the two individuals. There exist three possible values for each segment: zero, one and two. Zero indicates both haplotypes in that segment are different between the two individuals, two indicates both haplotypes in that segment are the same between the two individuals and one indicates only one of the haplotypes is the same between the two individuals.

### 2.3 Protecting privacy during identification of relatives

In order for individuals to securely compute the symmetric difference between their genomic sets, we define a sketch where we hash the value of each element in the genomic sets (*S_i_*). Let *K_i_* indicates the sketch of *i*-th individual and *k_ij_* indicates the *j*-th element of the *K_i_* that is obtained by hashing the *j*-th element of the *i*-th individual genome set.

$$k_{ij}=h_{24}\left(s_{ij}+r\right)$$

where *r* is a random binary number of size 100 that is referred to as the salt, and $h_{24}(.)$ is a collision-resistance hash function that returns the first 24 bits. One of the main properties of the elements in the secure set is that the similarity between two chunks is preserved. If two segments differ in one base pair their corresponding elements in the secure set differs due to the hash function.

Collision-resistance hash function has two main properties: first, collision-resistance hash function is one-way function. Second, finding distinct values which have the same hashed value is hard. We consider function *f* to be a one way function such that given *x* computing *f*(*x*) is easy. However, given the *f*(*x*) computing the *x* is hard. It is worth mentioning two segments obtained from the same genomic position in the genome for two different individuals that differ in one base pair have a different sketch element. Thus, reverse engineering the genome given the secure set is extremely hard based on the hardness of inverting one way functions.

However, using the sketch for identification leaks information. We can compare the sketch of other individuals with our own sketch to detect which genome segments are similar. Thus, this results in the leak of information. We use the sketch as the private key and use the improved version of the Juels–Sudan construction (Dodis *et al.*, 2008; Ishai *et al.*, 2011) that uses list decoding, followed by a hash check to generate a secure sketch that is used as public key for individuals.

Using the above encoding, each individual is represented by a set containing 24-bit elements. Individuals are related if they share at least ‘*t*’ of their elements. We can then use the secure set comparison from Section 2.1 to allow individuals to identify their relatives without requiring them to release their genomes.

The amount of entropy in ‘fuzzy’ encryption is bounded by $\frac{t^{2}}{s}$ where *t* is the number of elements that are in common between the sets sand *s* is number of chunks. In the case of human $s=\frac{3 000 000 000}{30 000}=100 000.$ Although computing the exact entropy of the human genome needs enormous number of individuals, He *et al.* (2013) show that the approximate amount of entropy in the human genome is much higher than $\frac{t^{2}}{s}.$ More detail is provided in Appendix A.

### 2.4 Haplotype encoding independent of genome builds

The encoding mentioned in Section 2.2 depends on the genome build that is used to call variants. Thus, individuals using different genome builds are unable to compare their sets. In this section we propose a new encoding which makes the encoding independent from the genome build which is used to call the variants. Our encoding is based on the observation that variant positions are typically identifiable using the 500-bp flanking sequence and the number of variants which differ in flanking sequence between different builds is extremely low.

In this encoding each segment is of size 30 000 bp and each segment starts from a known common SNP in the dbSNP (http://www.ncbi.nlm.nih.gov/SNP/). Then, for each variant in the segment we consider the flanking sequence of length 500 bp around the variant. Virtually all common SNPs have been identified in the HapMap and 1000G projects. We concatenate all the flanking sequences around each variant in a segment to represent the segment uniquely. Then, the collision resistance hash function is applied as described above to generate elements of the set.

### 2.5 Generating simulated data

In order for us to evaluate our method we must generate realistic simulations. We generate simulation by randomly mating individuals and generating a pedigree using a recombination rate of 10^−^^7^.

Since sequence errors and phasing errors affect the amount of matching in real data, for our simulations to be valid, we must use similar error rates. We utilize our real data to estimate the effect of these errors on matching in order to guide our simulations as follows. We first generate simulations without any error rates and compute the amount of matching for siblings unrelated individuals in real data compared to our simulated data. We then increase the error rate until the amounts of sharing are comparable and then utilize these parameters in our simulations.

## 3 RESULTS

### 3.1 Simulated data

In order to assess the performance of our method, we generated simulated data for different levels of relatedness using the 1000 genomes data. We used the LWK population which consists of 116 individuals. Among these 116 individuals 19 individuals have cryptic relationships that are removed from our data-generating process, and we used the remaining individuals as the founder individuals. In the first step, we used the founder individuals to generate offspring by randomly mating the individuals. Moreover, for simplicity we assume there exist no polygamy in the simulated data, thus each individual is mated with only one individual. In the next step, we use the generated offsprings to generate offsprings of the next generation by pairing together unrelated individuals from the current generation. We continue to generate new offsprings until we have sufficient number of distant relatives. In our case, we generated 10 generations from the founder individuals. Using this data we can check different levels of relatedness such as sibling, first-degree cousins, and second-degree cousins and up to sixth-degree cousins. We utilized a recombination rate of 10^−^^7^. We utilized a sequencing-error and phasing-error rate which is consistent with what we observe as the effect of errors on the amount of matching compared to what is expected in real data as we describe in Section 2.

**Fig. 2.**
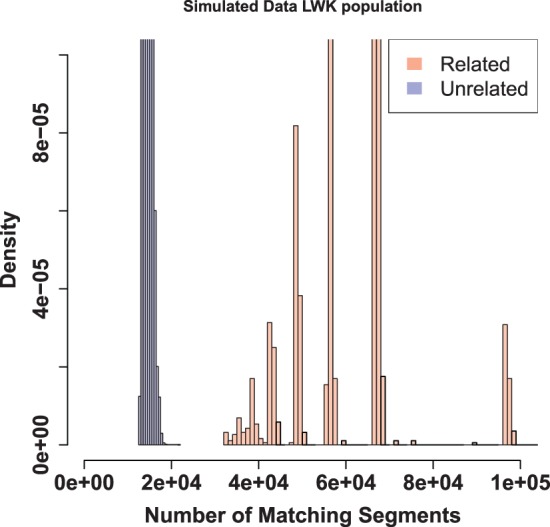
There exists a clear separation between the related and unrelated individuals. We use the LWK population from the 1000 genomes data as the founder and we use the cut-off of 25 390 segments to distinguish the related and unrelated individuals

We compute the similarity score for each pair of individuals using our encoding. We show there exists a separation between the related and unrelated pairs of individuals which is shown in Figure 2. We set the cut-off to 25 390 segments to separate the related individuals from unrelated individuals. In Appendix A we describe a principle way to select the cut-off.

**Fig. 3.**
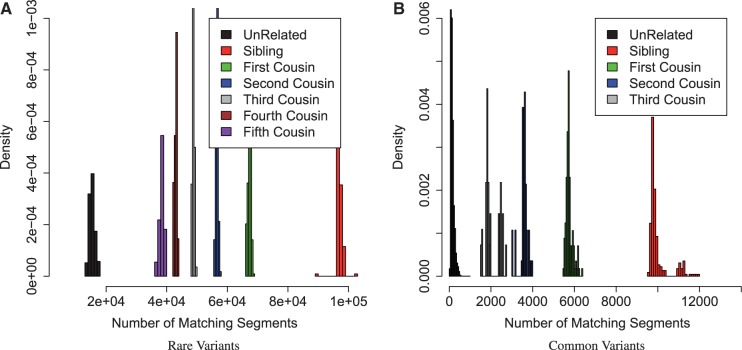
The histogram of the number of matched segments between different individuals in the simulated data. We used the set of unrelated individuals in the LWK population from the 1000 genomes data as the founder. Panel (**A**) indicates our method which uses the rare variants to detect the relativeness between the different individuals and panel (**B**) indicates the result of the method proposed by He *et al.* (2013). Thus, utilizing the rare variants, we can detect up to fifth-degree cousin as opposed to the third-degree cousin

Figure 3A indicates the histogram of similarity scores for different individuals. All pairs of individuals that have the same relationship are shown with the same color in the histogram. There exist a separation between the number of segments shared between related individuals compared to unrelated individuals, we set the cut-off to 25 390 segments to separate the related individuals from unrelated individuals. This result indicates that we can easily distinguish up to fifth-degree cousins using the rare variants. We note that in a previous approach, He *et al.* (2013) were able to distinguish only up to third-degree cousins which only utilize the common variants. The result of common variants is shown in Figure 3B.

We run our method to generate the secure sketch (public key) for each simulated individual and then each individual uses the secure sketch of another individuals and compare to its own sketch (private key). As expected, for each pair of individuals that are related, the program terminates successfully. However, for unrelated pairs of individuals the program fails.

We use another population from the 1000 genomes to generate simulated data using the same process to make sure our results are not specific to only one population. We use the Mexican Ancestry in Los Angeles, California (MXL) population. The MXL consist of 69 individuals where nine individuals have cryptic relationships. We removed the cryptic-related individuals so that the founders are unrelated. We observe there exists a separation between the related and unrelated using our method of comparing sets. We can detect up to fifth-degree cousins using our method. The results are similar to the LWK population and for the sake of space we did not show the results.

### 3.2 Real data

In order to assess the results of our method we used the 1000 genomes data. Although the 1000 genomes data consist of unrelated individuals, there exists three populations that contain cryptic (not known before sequencing) relationships. These three populations are African Ancestry in Southwest (ASW), and LWK. We used the final phase of data. The ASW population consists of 66 individuals where 10 individuals have cryptic relationships. The LWK population consists of 116 individuals where 19 individuals have cryptic relationships. The cryptic relationships in this data are parent–child, sibling or second-order relationships.

**Fig. 4.**
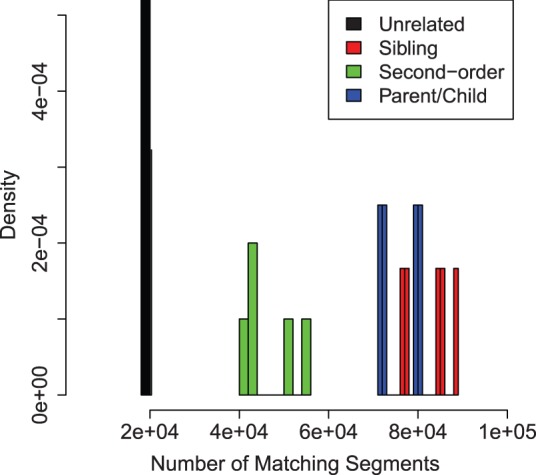
The histogram of the number of matched segments between different individuals in the 1000 genomes data. We used the ASW and LWK populations. For each pair of individuals we count the number of segments that are exactly match. We can use a cut-off of 25 390 segments to distinguish between the related and unrelated individuals in this dataset

In order to detect if two individuals are related or not there exist series of methods, the standard method is KING method (Manichaikul *et al.*, 2010). In this work we use a simpler idea which can be used to build a secure protocol. We divide the genome to segments of length 30 000 bits. Then, for each pair of individuals we count the number of segments which are identical and then use a threshold to distinguish between related and unrelated individuals. As shown in Figure 4 there exists a clear separation between the related and unrelated individuals based on the number of matched segments. Thus, the threshold of 25 390 number of segments can discriminate the related and unrelated individuals.

We run our method to generate the secure sketch (public key) for each individual in the 1000 genomes data. Then, each individual uses the secure sketches of other individuals and compare it with their own sketch (private key). As expected, for each pair of individuals that are related, the program terminates successfully. However, for unrelated pairs of individuals the program fails.

In order to check if the new encoding mention in Section 2.4 works, we used the known list of SNPs from Hg18 and Hg19 obtained from the HapMap project. For each SNP we consider 500-bp sequence around the SNP in both builds of Hg18 and Hg19. Then, we used the SSHA-256 to hash each string (1000 bp) and compared the hash value for the same SNPs in the two different builds. In our experiment we observed only 0.002 fraction of the SNPs will not have the same hash value. Meaning only 0.002 of SNPs are not mapped to the right SNP position when two different genome builds are used. As a result, the majority of SNPs are mapped to the same flanking sequence when moving from Hg19 to Hg18. Thus, the encoding which utilizes the flanking sequence can easily use a different genome build to generate keys to be compared with the other individual’s public key that was generated using a different genome build.

## 4 DISCUSSION

Sequencing technologies have made personal genomics possible and many companies are providing information about ancestry and health of individuals by utilizing genetic data. However, to obtain these information, each individual has to share their genomic data. The sharing of genomic data raises privacy issues.

One solution to the privacy issue is to use a trusted third party for detecting relatedness, however, individuals may not feel comfortable to share their genetic data with a trusted party for detecting related individuals. In this article, we demonstrate detecting the relatedness between two individuals where both individuals have access to their genetic data and no third party is needed.

Recently, He *et al.* (2013) have proposed a secure method for detecting the genetic relatives using genotype data. This method uses the ‘fuzzy’ encryption. A limitation of He *et al.* (2013) is that only previously know variants which are common can be used in the method. Unfortunately, common variants are not as nearly as informative for identifying relatives as rare variants which are typically shared with only close family members.

In this work, we provide a secure method for individuals to detect the genetic relatives from sequencing data without exposing any information about their genomes that utilizes both common and rare variants and through simulated data, we demonstrate, we can detect up to fifth-degree cousins. We also show in two populations from the 1000 genomes data that contains cryptic relationships, our method can detect these individuals. Our method also utilized an encoding that allows us to compare individuals who utilized different genome builds for calling their variants. Thus, genomes encoded using today’s genome build can be used to detect relatives called using future builds.

The input to our method is the phased haplotypes, in the case we have unpashed data, we phase our data using an existing method (Browning and Browning, 2007; Li,Y. *et al.*, 2010; Scheet and Stephens, 2006); Stephens and Scheet, 2010). We phased the individuals using a reference dataset of individuals which did not contain any individuals that are related to the ones we are phasing. We note that sequencing errors and phasing errors decrease the amount of segment matches between related individuals because an error in a segment that matches will appear as a segment that does not match. Our experiments over real data already implicitly take into account the sequencing and phasing errors because any errors decrease our observed amount of similarity among related pairs. As sequencing technologies mature and the error rates decrease, we expect that the number of matches between related individuals will increase accordingly.

## APPENDIX A

### A1 SEPARATION CUT-OFF BETWEEN RELATED AND UNRELATED INDIVIDUALS

In this section we describe a principled way to select a cut-off to separate the related from unrelated individuals. Using real data we observe the number of segments shared between unrelated individuals follows a normal distribution $N(\mu ,\sigma^{2})$ where the mean of the distribution is 19 325 and the standard deviation is 1080. Supplementary Figure S1 illustrates the QQplot of the number of matched segments between each pair of unrelated individuals in LWK population. Unfortunately, the real data lack sufficient number of related individuals to observe if the number of segments between related individuals follows a normal distribution or not.

Given that the number of shared segments for unrelated individuals follows a normal distribution $(X∼N(\mu ,\sigma^{2})),$ we select a cut-off value of *c* such that the probability of observing a value >*c* for the number of matched segment in unrelated individuals is extremely small such as 1*e* − 8.

P(X≥c)≤1e−8

Thus, in our real data we set the cut-off to 25 390 (*c* = 25 390).

### A2 IMPROVED JUELS–SUDAN CONSTRUCTION

In more detail, the idea of a secure sketch is based on the notion of an *error correcting code* (ECC) [Blahut (1983), Van Lint (1982) provide good introductory treatment of the theory or error correction]. An ECC is used to provide a reliable means of communication over noisy channels. Here, we provide a very brief and simplified overview of ECC that is sufficient for our purposes. For positive integers *n*, *k*, *d*, an (*n*, *k*, *d*) ECC is a *k*-dimensional subspace of an *n*-dimensional vector space. Each element of the *k*-dimensional subspace is called a *codeword*. The parameter *d* specifies the *distance* of the code, which means that the Hamming distance (the Hamming distance between two *n*-dimensional vectors is the number of co-ordinates where they differ) between any two code words is at least *d*. Thus intuitively, the distance of a code is a measure of how ‘spread-out’ the code words are in the *n*-dimensional space. Finally, the ECC comes with a mechanism to ‘correct small errors’. This means that given a codeword *v*, if we change a small number of coordinates of *v* to get a vector *w*, then there exists an algorithm that on input *w*, outputs the ‘correct’ codeword *v*. Formally, an ECC comes with an efficient *Decoding Algorithm*, which works as follows: given any *n*-dimensional vector *w* as input, if there exists a codeword within distance *d*/2 of *w*, then the decoding algorithm outputs that vector, otherwise, it outputs an error message specifying that decoding failed. Note that as the distance of the code is *d*, there can be at most a single codeword within a distance *d*/2 of any vector *w*. This is called unique decoding.

The Juels–Sudan construction that we use from Dodis *et al.* (2008) is based on a particular kind of ECC, called the Reed–Solomon code. We first give a brief overview of the Reed–Solomon construction, and then describe the Juels–Sudan construction. An (*n*, *k*, *d*) Reed–Solomon code is a particular kind of ECC that is defined as follows: fix a finite field *F* (in our case, the field *F* will be the Galois field $GF(2^{24})),$ and consider the *n*-dimensional vector space *F^n^*. To define the *k*-dimensional subspace of code words, we begin by fixing a sequence of *n* points $(a_{1},\ldots ,a_{n}),$ where each *a_i_* is an element of *F*. The subspace of code words is obtained by evaluating all the degree *k* – 1 polynomials (over *F*) on the points $(a_{1},\ldots ,a_{n}),$ i.e. let f(·) be a degree *k* – 1 polynomial whose coefficients are elements of *F*. Then the corresponding code word is $(f(a_{1}),\ldots ,f(a_{n})).$ The code word subspace consists of the evaluations of all degree *k* – 1 polynomials. It follows from elementary algebra that the distance of the Reed–Solomon code is d=n−k+1. The details of the decoding algorithm can be found in Blahut (1983), Van Lint (1982).

Now we are ready to describe the improved Juels–Sudan construction from Dodis *et al.* (2008). Recall that the genome is represented as a set of 24-bit strings, which we take to be elements from the field $GF(2^{24}).$ Let $s_{1}=\{w_{1},\ldots ,w_{n}\}$ be such a set. Our task is to convert the genome sketch *s*_1_ to a ‘secure sketch’ *ss*_1_, which satisfies two properties: (i) the secure sketch should not reveal too much information about *s*_1_, and (ii) given the genome sketch $s_{2}=\{v_{1},\ldots ,v_{n}\}$ of another individual and the secure sketch *ss*_1_ of the first individual, we should be able to determine if the two individuals are related or not. The Juels–Sudan algorithm uses algebraic techniques to achieve this.

One of the main ideas of the Juels–Sudan construction is to represent the genome sketch as a polynomial. In particular, we first construct a polynomial *p*(*x*) whose roots are the *w_i_*s; that is $p(x)=\prod_{i=1}^{n}\left(x-w_{i}\right).$ Note that anyone who knows *p*(*x*) can obtain the entire genome sketch by simply finding the roots of *p*(*x*). Thus, in particular, we can not use *p*(*x*) itself as the secure sketch (as it reveals too much information about the genome). Instead, the idea is to reveal only a small part of the polynomial *p*(*x*), and reconstruct the rest using error correction. This is done as follows: *p*(*x*) is split into two polynomials $p_{\text{high}}(x)\;\text{and}\;p_{\text{low}}(x).$ Polynomial $p_{\text{high}}(x)$ is a degree-*n* polynomial that matches *p*(*x*) in the *ℓ* highest coefficients, and all the other coefficients are 0 (here, the parameter *ℓ* will be determined later). The polynomial $p_{\text{low}}(x)$ is a degree-*n* − *ℓ* polynomial that matches with *p*(*x*) in the n−ℓ smallest coefficients. Thus, $p(x)=p_{\text{high}}(x)+p_{\text{low}}(x).$ Only the polynomial $p_{\text{high}}(x)$ is released in public. To complete the scheme, we have to show two things: (i) revealing $p_{\text{high}}(x)$ does not reveal too much information about the genome sketch, and (ii) given $p_{\text{high}}(x),$ and the genome sketch of another individual, we can find out if there is a match or not.

We first describe how matches are determined. Let $\left\{v_{1},\ldots ,v_{n}\right\}$ be the genome sketch of another individual. Note that if we can reconstruct the polynomial *p*(*x*), then it is easy to check if there is a match or not (as *p*(*x*) contains all information about the sketch $\{w_{1},\ldots ,w_{n}\}).$ As $p_{\text{high}}(x)$ is publicly available, our task is to reconstruct $p_{\text{low}}(x).$ First, note the following mathematical fact: as *w_i_* is a root of *p*(*x*), we have, $p(w_{i})=0,$ which implies that $p_{\text{high}}(w_{i})+p_{\text{low}}(w_{i})=0$ or $p_{\text{low}}(w_{i})=-p_{\text{high}}(w_{i}).$ This implies that even though we do not have $p_{\text{low}}(x),$ we can evaluate it on *w_i_* given $p_{\text{high}}(x),$ which is publicly available. Further, if we can evaluate $p_{\text{low}}(x)$ on large enough number of points, then we can reconstruct $p_{\text{low}}(x)$ using elementary algebra (by a process called polynomial interpolation). However, we do not have access to the *w_i_*s, but only to *v_i_*s. But if the individuals are related, then the genome sketches of the individuals are close together, which means most of the *w_i_*s are the same as *v_i_*s. Thus, if we evaluate $p_{\text{high}}\text{(x)}$ on the *v_i_*s, we obtain a ‘noisy’ version of the evaluations of $p_{\text{low}}(x).$ And this can now be corrected using error correction. In particular, we construct the *n*-dimensional vector $(p_{\text{high}}(v_{1}),\ldots ,p_{\text{high}}(v_{n})),$ and run the decoding algorithm of the Reed–Solomon code on it. If the two genome sequences are close by, then this algorithm outputs closest code word, which is $(p_{\text{high}}(w_{1}),\ldots ,p_{\text{high}}(w_{n})),$ from which $p_{\text{low}}(x)$ can be reconstructed.

Now we come to the first point above, namely that revealing $p_{\text{high}}(x)$ does not reveal too much information about the genome. Clearly, the amount of information released depends on the value of *ℓ*; the smaller the value of *ℓ*, the smaller the amount of information released. On the other hand, we can not make *ℓ* too small, as then we will not have enough information to decode (note that we are trying to reconstruct an *n* − *ℓ*-degree polynomial from *n* noisy points). Let *t* be the threshold for matching, i.e. if two individuals are related, then there genome sketches have at least *t* points in common. Then, to minimize the value of *ℓ*, we need to find the largest degree of the polynomial $p_{\text{low}}(x)$ that can be correctly decoded given *n* points, with threshold *t*. For the Reed–Solomon code with unique decoding, this turns out to be *t*, and thus the remaining entropy is equivalent to *t* field elements.

Unfortunately, the way we have described the Juels–Sudan scheme above does not work for our application. The reason is that unique decoding of Reed–Solomon requires that the agreement be very high, as compared to the size of the genome sketch. However, in our application, even if the individuals are related, the agreement can be very small. Thus, we move to a more sophisticated error correction scheme called ‘list-decoding’ for Reed–Solomon codes. The main advantage of list-decoding over unique decoding is that it can tolerate very small agreement thresholds also. The scheme remains essentially as we have described so far, except that in the reconstruction step, instead of using unique decoding to reconstruct $p_{\text{low}}(x),$ we use the list-decoding algorithm from Guruswami and Sudan (1998). The remaining entropy in this case turns out to be $t^{2}/s$ field elements. In the case of human $s=\frac{3 000 000 000}{30 000}=100 000.$ Although computing the exact entropy of the human genome needs enormous number of individuals, He *et al.* (2013) show that the approximate amount of entropy in the human genome is much higher than $\frac{t^{2}}{s}.$
